# An Epistle upon Yellow Fever

**Published:** 1858-11

**Authors:** 


From N. O. Med. & Sur. Journal.

### *An Epistle upon Yellow Fever*

RAYMOND, MISS., August 11, 1858.

DR. BENNET DOWLER, *New Orleans, La:* Dear Sir,—I hope you will excuse me for taking the liberty of asking you a few questions upon the subject of the treatment of yellow fever, I know that your time is very much occupied with your various duties, and, consequently, I feel afraid that I am drawing too heavily upon your kindness in requesting you to give me the desired information. If you can do so with convenience, you would oblige me by giving me the general course of treatment pursued by those most successful in the treatment of the disease in question.

Are the patients confined under blankets, to induce and keep up a perspirable state of the skin? or are they allowed simply covering enough to make them comfortable, as in the treatment of other diseases? etc., etc.

Very respectfully, your obed't serv't,

GEO. LATIMER, M. D.

---

EVERY where in the medical world, and by every body in it, is felt one prevailing hope, one great desiderated finality, namely, a clear, precise, and certain knowledge of the method by which diseases, whether isolated, endemic, or epidemic, may be cured. In all ages since the eating of

> "The fruit
> Of that forbidden tree, whose mortal taste
> Brought death into our world and all our wo,"

sages and charlatans, the wise and the unwise, have clearly seen that this is the first, last, only aim of the healing Art, which Hippocrates and his successors for more than two thousand years have labored to achieve;

> "Which kings and prophets waited for
> And sought, but never found."

Yet the experience of the ancients for more than two thousand years has been virtually, almost entirely lost, because they have not transmitted to their successors the precise diagnostics and natural classification of diseases, together with their pathology and therapeutics.— What satisfactory information is derivable from Hippocrates' account of his visit to the son of Parion, a fever patient, who died on the 120th day, near the Temple of Diana? or what can bc learned in the case of Pythion, who lived above the Temple of Hercules, and whose fever did not reach a crisis until the 100th day? or from the case of Heropythus, whose fever proved fatal on the 10th day? Perhaps the first two cases were typhoid such as Louis and Chomel have described, and such as I have seen and treated in Virginia; cases which have run on to the 100th day. Who can tell? Had Hippocrates given the diagnosis, pathology and treatment of these and many other cases of fever to which he alludes, the study of these, and their comparison with febrile maladies in every æra and in every place, would like a gushing mountain spring, have flowed in a continuous and increasing stream down the declivities of time, irrigating the fields of science.

In reality, medical, not less than cosmical phenomena, may be uniform, fixed and certain ; yet, owing to our imperfect knowledge of the complete history of the former, they appear unstable, changeful, irregular, and sometimes altogether uncertain. For the individual student, medicine must be perpetually reinvestigated, nay, re-created out of the past and the present. The labors and the thoughts of others, will, for him, supersede neither the necessity for experimental labor nor the necessity for laborious thinking.

Would not the same medicine, it may be asked, which cured a Greek, or Roman, cure a Louisianian, or a Mississippian ? Undoubtedly ; provided the cases be precisely alike, parallel in every condition and direction. But how is this identity to be determined and made manifest beyond mistake ? Perhaps no written description can be given of the normal, not to mention the pathological appearance and physiognomy of any person in New Orleans so as to be recognized by a person in Raymond, Miss. It is still more difficult to portray in a recognizable form the differential diagnosis of diseases and their varying stages and rapid metamorphoses occuring in the same epidemic, for which new combinations of remedial measures may be required, and in which judgment and personal observation are involved. For example, there is in a comparatively limited number of yellow fever cases at the very onset, acute pains throughout the body, as if the pains of dengue, rheumatism, and choleraic spasms, were concentrated in one individual. A stout man, otherwise courageous, rolls and screams, and annoys his neighbors. A solution of the sulphate of morphia, if not contraindicated by some condition of the brain, which, however, is not very likely to happen, will, in such a case, afford surprising, almost immediate relief, without interfering with ulterior curative measures. The beneficial effects of opiates are not, however, restricted to such cases alone.

The removal of pain is a gain by no means to be despised. Pain is an element of disease of great significance whether it originate in the dynamics, or in structural changes of the economy. Its elimination augurs favorbly for the ulterior treatment by either the Doctor or the Doctress, *Vis Medicatrix Naturæ.*

But neither my time nor the remaining space of the Journal will admit of going into the remedial plans and measures which I prefer in the treatment of Yellow Fever.

But it may be said—"we asked for bread, and lo! we have received a stone." Not exactly so; for if the gentleman who does me the honor to ask "whether the patients are to be confined under blankets, etc., etc., etc., will look into this journal, Vols. XI and XIII, particularly the latter, he will find my views expressed at length upon this part of his inquiry, not only physically, and physiologically, but pathologically and therapeutically, and, without going back beyond the times of the Cæsars for authorities, lo! is it not written in Celsus, (*De Medicina*, l. iii. c. vii.) that at the onset of an ardent fever, even in advance of all internal medication, the patient must be *refrigerated* with oil and water, and be put in a well ventilated chamber where he may have abundance of pure air; he is to be covered lightly, not suffocated, literally strangled, by bed clothes, nor *a fortiori* by blankets and feather beds: "*Si vero ardens febris extorret, nulla medicamenti danda portio est; sed in ipsis accessionibus oleo* (the oil may safely be omitted) *et aqua* REFRIGERANDUS EST, *que miscenda manu sunt, donec albescant; eo conclavi tenendus, quo* MULTUM ET PURUM AEREM TRAHERE POSSIT; NEQUE MULTIS VESTIMENTIS STRANGULANDUS, SED ADMODUM LEVIBUS TANTUM VELANDUS EST." I venture to think that ætiology, physiological chemistry, clinical experience, common sense, and instinct, are on the side of the Roman Hippocrates.

There are several particulars in which physicians without experience in yellow fever are very liable to be mistaken. For example, the diagnostication of this malady in its inception is often difficult for the most experienced, though its advanced stage is easily recognized even from written or verbal descriptions. The prognosis is also most illusory to the uninitatied. It is for such, scarcely possible to admit, that a patient with little or no pain, having a full, soft and regular pulse, etc., will nevertheless, die the same or following day.

To a well informed practitioner who may never have witnessed yellow fever, there is but little mystery in it not common to other fevers, so far as treatment is concerned; for this, whether right or wrong is generally simple, being with most practitioners directed to the repression or removal of such prominent symptoms as arise during the progress of the malady. Those who look forward to the eventualities or to symptoms which must be anticipated and prevented, and, who adopt as fundamental a systematic treatment conformably to their theory of the pathology of the disease, without waiting for actualities, will accomplish their purpose best, if at all, by avoiding, as they now generally do, heroic measures; for, notwithstanding this is formidable fever, yet with rare exceptions neither antiphogistics nor stimulants are now pushed to a formidable extent upon the merely physical principle or false analogy that in therapy the greater force will overcome the inferior, heavy battalions, weak ones. An apothecary showed me a prescription directing an ounce and a quarter of quinine to be administered by the mouth and by enemata to a gentleman, the day before death from yellow fever; another, assured me that an ounce of this drug in solution was applied to a man's skin in this disease, without having produced any appreciable effect. As to myself I have seen what I have seen, that is, a good deal of quininism. * * *

Now, if calomel be deemed in the early stage of this disease, a good febrifuge aperient, and an alterative promoter of the secretions and excretions—if quinine, opium, and diffusible stimulants be accepted as alterative, tonic, calmative, nervine, diaphoretic, and febrifuge, small doses are preferable. For example, if quininism or saturation be deemed essential, two grain doses will effect this end causing the characteristic affection of the organ of hearing, without the impending dangers to vision, to the brain, etc., which sixty grain doses occasionally produce. At least, such is my opinion.

Without having used large doses of opium in my own practice, yet from what I have seen and learned of the practice of others, it appears that this potent drug in large doses is not only tolerated, but often highly beneficial as a febrifuge, sedative and diaphoretic, etc., in not a few cases of yellow fever, particularly in the extensive practice of Dr. M. Morton Dowler, during the present and several former epidemics of New Orleans. Without his permission or knowledge, I will here say for the information of my Raymond correspondent, that Dr. M. M. D. is now using the drug aforesaid, together with refrigerations of the surface in the hot stage, with cold vinegar, whiskey, and water.

In conclusion, may I not allude to a stumbling-block, or rather a truism now much in vogue with some writers, namely, that yellow fever and not a few other fevers and maladies are *self-limited?* True! Everything is limited except eternal duration and infinite space. Yellow fever is neither infinite nor eternal. It is limited, but its limits are very irregular whether fixed by its self-determination or by the determination of another. If yellow fever be *self*-limited, and have a will of its own, it is very *selfish* in not making its limits known; for unless its phenomenal manifestations be understood contrarywise to their actual appearance, neither its symptoms nor duration are uniform, but contingent, being not only variable when left to themselves, but more or less controllable for good or evil by medical treatment. Even small-pox, measles, scarlatina, erysipelas, and several other diseases which are the most uniform in character and duration, and the least amenable to medication, are nevertheless, in all probability often cut short or modified both as it regards their tendency to be protracted, or to end fatally. Suppose the natural tendency of yellow fever is is to run its course, ending in death or convalescence in a week, but in certain cases being attended with unusual symptoms and complications more or less controllable by art; again, suppose that this tendency, these unfavorable conditions prolonging the disease and giving it intensity so as to end fatally at last, should be changed, is it not reasonable to suppose that this control if timely exercised, would often shorten or prevent this otherwise persisting and fatal tendency inherent in the original malady, or arising accidentally during its progress? To control symptoms is to control results and duration.

While, on the one hand, bad treatment may accelerate the progress and fatal termination of yellow fever; on the other hand good treatment may often shorten its duration, and even cure the patient. It may be well to throw a little cold water on the too ardent zeal of those who profess ability to cut up the disease root and branch, forth with; but the alleged doctrine of the self-limitation of fevers as a reason for not attempting to control or cure them, is in itself an affected hypothesis, being withal calculated to bring despair to the practitioner and to fill the public mind with an unwarrantable distrust in regard to the efficiency and utility of the medical profession. The physician should not accept the captivating phrases of medical skeptics, concerning *self-limitation* as a safe theory either in medical or ethical conduct with reference to persons and families who commit under God, their health and lives to the hand of medical men.

This doctrine of self-limitation, which is almost synonymous with therapeutic inaction, generally, is accepted neither by the surgeon nor obstetrician; these latter, not the blind diseases and self-murderous aberrations of the disordered economy, undertake themselves to do the work of limitation.

BENNET DOWLER.

